# Protective role of estrogen against excessive erythrocytosis in Monge’s disease

**DOI:** 10.1038/s12276-020-00550-2

**Published:** 2021-01-20

**Authors:** Priti Azad, Francisco C. Villafuerte, Daniela Bermudez, Gargi Patel, Gabriel G. Haddad

**Affiliations:** 1 Department of Pediatrics, Division of Respiratory Medicine, University of California, San Diego, La Jolla, CA 92093 USA; 2 Laboratorio de Fisiologia del Transporte de Oxigeno/Fisiología Comparada, Facultad de Ciencias y Filosofía, Universidad Peruana Cayetano Heredia, San Martin de Porres, Lima 31, Peru; 3 Department of Neurosciences, University of California, San Diego, La Jolla, CA 92093 USA; 4 Rady Children’s Hospital, San Diego, CA 92123 USA

**Keywords:** Haematopoietic stem cells, Myeloproliferative disease

## Abstract

Monge’s disease (chronic mountain sickness (CMS)) is a maladaptive condition caused by chronic (years) exposure to high-altitude hypoxia. One of the defining features of CMS is excessive erythrocytosis with extremely high hematocrit levels. In the Andean population, CMS prevalence is vastly different between males and females, being rare in females. Furthermore, there is a sharp increase in CMS incidence in females after menopause. In this study, we assessed the role of sex hormones (testosterone, progesterone, and estrogen) in CMS and non-CMS cells using a well-characterized in vitro erythroid platform. While we found that there was a mild (nonsignificant) increase in RBC production with testosterone, we observed that estrogen, in physiologic concentrations, reduced sharply CD235a^+^ cells (glycophorin A; a marker of RBC), from 56% in the untreated CMS cells to 10% in the treated CMS cells, in a stage-specific and dose-responsive manner. At the molecular level, we determined that estrogen has a direct effect on GATA1, remarkably decreasing the messenger RNA (mRNA) and protein levels of GATA1 (*p* < 0.01) and its target genes (*Alas2*, *BclxL*, and *Epor*, *p* < 0.001). These changes result in a significant increase in apoptosis of erythroid cells. We also demonstrate that estrogen regulates erythropoiesis in CMS patients through estrogen beta signaling and that its inhibition can diminish the effects of estrogen by significantly increasing HIF1, VEGF, and GATA1 mRNA levels. Taken altogether, our results indicate that estrogen has a major impact on the regulation of erythropoiesis, particularly under chronic hypoxic conditions, and has the potential to treat blood diseases, such as high altitude severe erythrocytosis.

## Chronic mountain sickness: Female hormone provides protection

The hormone estrogen protects against chronic mountain sickness (CMS) in pre-menopausal women and may prove valuable in treating the condition. People who live for years in high altitude mountain regions are susceptible to CMS because of prolonged oxygen deprivation. One factor in CMS is an over-production of red blood cells, thickening the blood and increasing the risk of strokes and cardiovascular diseases. Men are more likely to suffer CMS than women, although cases spike in females after menopause. Gabriel Haddad at the University of California in San Diego, USA, and co-workers used a cell culture model of CMS to demonstrate that estrogen provides protection against the disease. Estrogen significantly alters the expression and activity of red blood cell-related genes to regulate red blood cell levels by controlling cell death mechanisms.

## Introduction

Chronic mountain sickness (CMS), or Monge’s disease, is a progressive and debilitating syndrome caused by chronic (years or lifetime) exposure to high-altitude hypoxia^1–9^. Globally, CMS is not a rare condition; it is estimated that there are >100 million people who live at high altitudes (>2500 m) and are at risk for developing CMS^4,7,8,10–13^. In this study, we take advantage of an “experiment in nature” in which human populations at high altitude in the Andes have adapted to chronic hypoxia, whereas other Andean individuals at the same high altitude do not seem to have adapted. These non-adapted individuals have CMS or Monge’s disease. Those who have adapted to high altitude and do not manifest disease are referred to as the non-CMS population. In addition to Andeans, two other large, high-altitude populations, Ethiopians and Tibetans, have been extensively studied^8,9,14–18^.

Excessive erythrocytosis (EE) is one of the critical traits of CMS, and this excessive pathobiologic response has deleterious effects since a high hematocrit would increase blood viscosity and reduce blood flow to hypoxia-sensitive organs (e.g., brain and heart), often resulting in myocardial infarction or stroke in young adults. From a phenotypic point of view, CMS patients suffer mostly from severe polycythemia along with neurologic and cardiovascular ailments^3,7,8,11,12,19,20^. Symptoms of CMS include headache, dizziness, breathlessness, palpitations, sleep disturbance, mental fatigue, confusion, pulmonary and systemic hypertension, heart failure, and polycythemia. Among all the signs and symptoms, the hematocrit remains a clearly defined quantitative trait and has been strongly linked to the disease, especially in the Andean population^3,7,8,11,12,19,20^.

One of the important epidemiologic observations about CMS in the Andes is that CMS affects mostly males and is rare in females^7,21–24^. There is also a sharp increase in CMS incidence in females after menopause^7,23,24^. This suggests the role of gender and sex hormones in altering the disease manifestation. Most studies, however, have focused on the basis for EE in males and have suggested that there is a correlation between testosterone levels and excessive erythropoiesis^25–29^, but these studies lack direct evidence or mechanisms that confirm the hypothesis^25–29^. To our knowledge, there have been no studies that clearly establish the role of sex hormones and the mechanistic basis for the remarkable sex differences in Monge’s disease, and no studies have attempted to understand the reason for the rarity of the disease in premenopausal females.

With advances in next-generation sequencing, major strides have been made to understand the genetic basis of hypoxia tolerance or sensitivity in high-altitude dwellers^2,8,18,30–37^. Our group and others have found a number of candidate genes that are differentially expressed when CMS are compared with non-CMS subjects. Others have corroborated our findings in this field, such as the role of the desumoylase SENP1, which has been confirmed by other groups^38–40^. Furthermore, using induced pluripotent stem (iPS)-derived cells from this unique Andean population, we have developed an in vitro model that mimics hypoxia-induced excessive polycythemia in CMS subjects^36^. Using our model system, we believe that we are in a position to further our cellular and molecular understanding of this gender difference in excessive erythropoiesis of high altitude.

## Materials and methods

### Patient samples and iPS cell lines

All study subjects (CMS and non-CMS) are adult males and females residing in the Andean mountain range in Cerro de Pasco, Peru, at an elevation of ∼4338 m. CMS patients fulfilled the diagnostic criteria for CMS or Monge’s disease based on the Qinghai CMS score, as described in detail in our previous studies^36,41^. Each subject signed an informed written consent under protocols approved by the University of California, San Diego and the Universidad Peruana Cayetano Heredia, Lima, Peru. iPS cell lines from CMS and non-CMS subjects have been generated and well characterized by our group^36,41^.

### RBC production using cytokines

RBCs were generated under normoxic as well as hypoxic conditions following our previously established in vitro platform based on the protocol of Duogay’s group^36,42^. Using this in vitro model and iPS cells, we have thoroughly studied erythroid differentiation using appropriate CD markers, as well as functional analysis of hemoglobin^36^. For the generation of RBCs from native CD34^+^ cells, the protocol from Duogay’s group was followed with the modification of the addition of a hypoxic regimen, as we have previously characterized this model in CMS and non-CMS subjects^43,44^.

### Hypoxia regimen and FACS analysis

Erythroid cells at the EB stage were cultured for 1 week at 37 °C in 5% CO_2_/air. After 1 week, these cells were transferred to a hypoxic incubator set at 37 °C, 5% O_2_, and 5% CO_2_ for 3 weeks. Subsequently, the cells were subjected to FACS analysis (FACSCanto Cell Analyzer (BD) using FACSDiva software, version 6.0; BD), and glycophorin A was used as a marker for the assessment of RBC production and maturation.

### Burst-forming unit-erythroid (BFU-e) assays

FACS-sorted iPS-derived CD34^+^ cells from CMS and non-CMS subjects were plated at a density of 10^5^ cells per 35-mm dish in MethoCult H4034 Optimum media with 2% fetal bovine serum. The dishes were incubated at 37 °C with 5% CO_2_ and 5% O_2_ for 14 days, at which time the colonies were scored for BFU-E colonies, and statistical *t* tests were performed to determine the significance of differences between the samples.

### Addition of sex hormones (testosterone, progesterone, and estrogen) during erythroid differentiation

(a) *Dose–response experiments*: Testosterone, progesterone, and beta-estradiol were purchased from Sigma and mixed with culture media at various concentrations extensively covering the known physiological ranges of these hormones. By evaluating a number of concentrations ranging from 5 to 100 nM (5, 10, 50, and 100 nM), a dose–response curve was generated for each hormone.

(b) *Time-course experiments*: Sex hormones were added at various time points during erythroid differentiation. Hormones were added during week 1, week 2, and week 3 at the EB stage of erythroid differentiation under hypoxia, as shown in the schematic below.



### Addition of estrogen alpha- and beta-antagonist

MPP (methyl-piperidino-pyrazole, an alpha-antagonist), THC (tetrahydrocannabinol, a beta-antagonist), and PHTPP (4-[2-phenyl-5,7-bis(trifluoromethyl) pyrazolo[1,5-a]pyrimidin-3-yl]phenol, a beta-antagonist) were purchased from Sigma and added to the cultures at a concentration of 10 nM (based on the pilot dose–response results).

### Addition of BclxL inhibitors

Specific BclxL inhibitors were purchased from Abcam (inhibitor #1, A-1155463; inhibitor #2, A-1331852) and added to the cultures at a concentration of 1 µM (based on the pilot dose–response experiment).

### Apoptosis assays

Apoptosis analysis was performed using the APC Annexin V Apoptosis Detection Kit with propidium iodide (PI) (BioLegend). The analysis was performed based on the manufacturer’s instructions. Briefly, the cells were washed twice with cold BioLegend Cell Staining Buffer and then resuspended in Annexin V Binding Buffer at a concentration of 0.5 × 10 cells/ml. We transferred 100 µl of cell suspension to a new tube and added 5 µl of APC Annexin V and PI solution. The cells were vortexed gently and incubated for 15 min at room temperature (25 °C) in the dark. Finally, 400 µl of Annexin V Binding Buffer was added to each tube and analyzed by flow cytometry using a FACSariaII System (BD Biosciences).

### Real-time PCR analysis for gene expression measurement

Erythroid cells at the EB stage were used after 1, 2, or 3 weeks in culture under hypoxia (with and without estrogen). RNA was isolated from EBs using RNeasy Mini Kit (Qiagen). Complementary DNA was produced from total RNA through RT-PCR using a Superscript Vilo IV System (Invitrogen). Real-time PCR was performed using a GeneAmp 7900 sequence detection system with POWER SYBR Green (Applied Biosystems). The primer sequences used were as follows:

GATA1-L, 5′-CCTGCTTTGTTGCCAATG-3′ and GATA1-R, 5′-CTGCTCCACT GTTACGGATAC-3′; VEGF-L, 5′-ATCTTCAAGCCATCCTGTGTGC-3′ and VEGF-R, 5′-CAAGGCCCACAGGGATTTTC-3′; EpoR-L, 5′-GCACCGAGTGTGTGCTGAGCAA-3′ and EpoR-R, 5′-GGTCAGCAGCACCAGGATGAC-3′; ALAS-2-L, 5′-ACAGTGCTGCCCAGTGCTTG-3′ and ALAS-2-R, 5′-TCCGACAGCATGAAGGGACA-3′; HIF1A-L, 5′-GAAAGCGCAAGTCTTCAAAG-3′ and HIF1A-R, 5′-TGGGTAGGAGATGGAGATGC-3′; HIF1B-L, 5′- CCCCGAAATGACATCAGATG-3′ and HIF1B-R, 5′-GTTCCCTTCTCCATCATCATC-3′; Bcl-xL-L, 5′-GCAGGTATTGGTGAGTCGGATCGC-3′ and Bcl-xL-R, 5′-CACAAAAGTATCCCAGCCGCCG-3′. The expression level of GAPDH was used to normalize the results, with the following primers: GAPDH-L, 5′-CTGGCATTGCCCTCAACGACC-3′ and GAPDH-R, 5′-CTTGCTGGGGCTGGTGGTCC-3′.

### Western blot analysis for quantification of protein levels

Proteins were isolated using standard protein isolation protocols with RIPA buffer and protease inhibitor cocktail (Abcam). Antibodies against GATA1, VEGF, and BclxL were obtained from Santa Cruz (M-20) and Abcam (ab69479, ab32370). In brief, 20 µg of lysate supernatant was separated by 10% sodium dodecyl sulfate-polyacrylamide gel electrophoresis and transferred to a nitrocellulose membrane. The blots were developed using enhanced chemiluminescent reagents (Bio-Rad Laboratories) and the ChemiDoc XRS+ molecular imager (Bio-Rad Laboratories).

### Lentiviral vectors and transduction of iPSCs to generate knockdown and overexpression (OE) cell lines

GATA1-OE, an expression-ready construct for the lentiviral system, was purchased from GE Healthcare. Estrogen-resistant GATA1-[f(GATA-1)-VP16]^45,46^ was generated by Vector Builder. Transduced cells were selected with 0.5 µg/ml puromycin (Sigma-Aldrich). The expression of each construct was verified by real-time PCR.

## Results

### Estrogen prevents hypoxia-induced RBC production at various erythroid stages

**Fig. 1:**
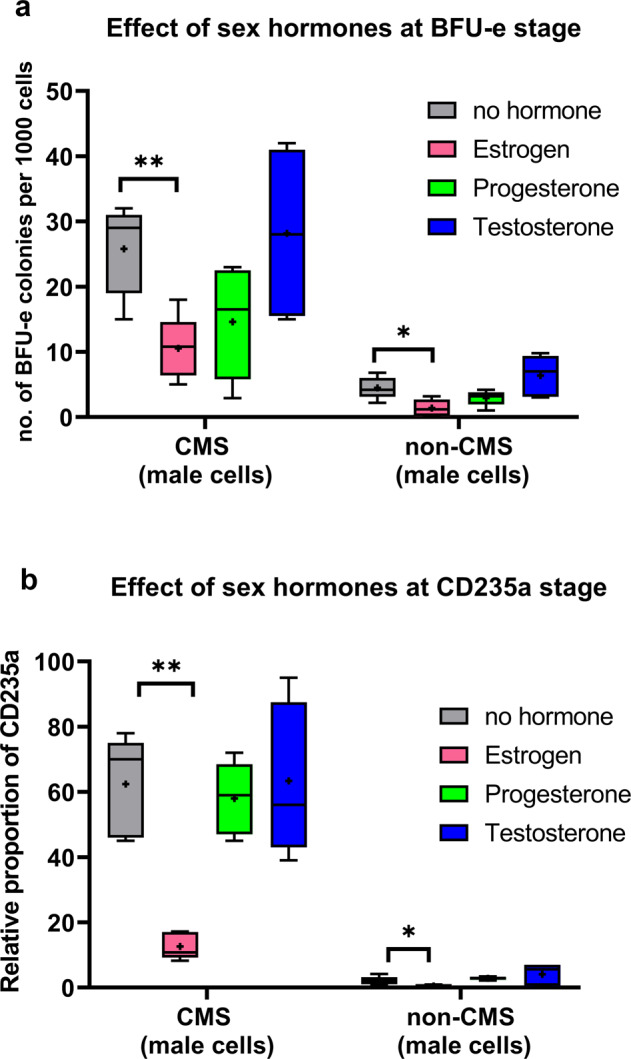
Effect of sex hormones (estrogen, progesterone, and testosterone) on erythroid cells from CMS and non-CMS males (at the BFU-e and CD235a stages). **a** The graph depicts the number of BFU-e colonies produced under hypoxia by iPSC-derived CD34^+^ cells from CMS and non-CMS individuals with the addition of the following sex hormones: (i) estrogen (pink bar), (ii) progesterone (green bar), (iii) testosterone (blue bar), and (iv) no hormone added (gray bar). Estrogen significantly (*P* < 0.05, *t* test) decreased BFU-e production in CMS cells. The number of subjects in each group is *n* = 4. “+” shows the mean of the values, and the horizontal line within each box denotes the median. **P < 0.01 and *P < 0.05. **b** The figure shows the effect of sex hormones on RBC production (as the relative proportion of CD235a^+^) in CMS and non-CMS cells under hypoxia with the addition of the following sex hormones: (i) estrogen (pink bar), (ii) progesterone (green bar), (iii) testosterone (blue bar), (iv) no hormone added (gray bar). Estrogen significantly decreases the proportion of mature RBCs (CD235a^+^ cells) in CMS cells. *P* value is <0.001 (*t* test). The number of subjects in each group is *n* = 4. “+” shows the mean of the values, and the horizontal line within each box denotes the median. **P < 0.01 and *P < 0.05.

**Fig. 2:**
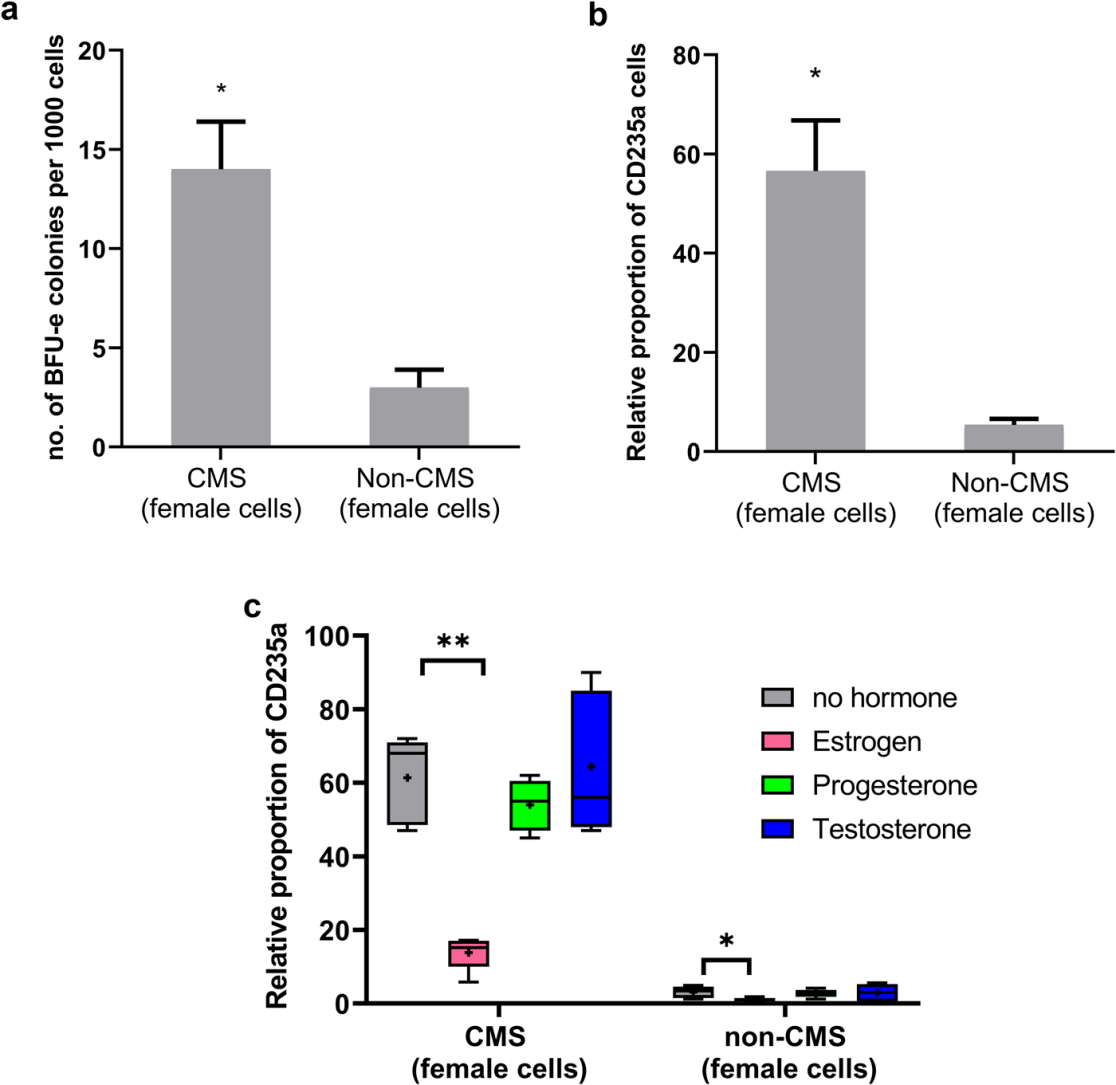
Postmenopausal erythroid cells from CMS and non-CMS females show differential responses to hypoxia, as shown previously in male cells. The addition of estrogen has a significant impact on RBC production in these female cells. **a** Hypoxic response of CMS and non-CMS postmenopausal female cells at the BFU-e stage. CMS cells show an excessive erythrocytosis response (EE) compared to that of non-CMS cells. This response is similar to that of male cells at the BFU-e stage. *P < 0.05 (*t* test). **b** Hypoxic response of CMS and non-CMS postmenopausal female cells at the CD235a stage. CMS cells produce excessive amounts of RBCs compared to non-CMS cells under hypoxia. *P < 0.01 (*t* test). **c** Estrogen reduces the excessive erythrocytosis phenotype in female CMS cells. The addition of estrogen significantly blunts the exaggerated response of CMS cells under hypoxia. The figure shows the effect of sex hormones on the RBC production (as the relative proportion of CD235a^+^) of CMS and non-CMS cells under hypoxia with the addition of the following sex hormones: (i) estrogen (pink bar), (ii) progesterone (green bar), (iii) testosterone (blue bar), and (iv) no hormone added (gray bar). Estrogen significantly decreases the proportion of mature RBCs (CD235^+^ cells) among CMS cells. *P* value is <0.001 (*t* test). The number of subjects in each group is *n* = 4. “+” shows the mean of the values, and the horizontal line within each box denotes the median. **P < 0.01 and *P < 0.05.

In our previous study, we have recapitulated the strong hypoxia-induced EE response of CMS cells in the dish^36^. We have also validated our results using native CD34^+^ cells and demonstrated the EE phenotype in CMS patients using both model systems^43^. In this study, we used this in vitro platform to study the influence of sex hormones on specific erythroid stages, such as on BFU-e as well as CD235a stage, under hypoxia in both CMS and non-CMS cells. Remarkably, we found that estrogen significantly decreases BFU-e colony production as well as the relative proportion of CD235a^+^ cells (more mature RBCs) in CMS male (Fig. 1) and female erythroid cells (Fig. 2). Figure 1 shows the effect of the addition of estrogen, progesterone, and testosterone on erythroid progenitors and CD235a^+^ RBCs in male CMS and non-CMS cells. In the CMS cells, the number of BFU-e colonies was reduced to half of what it was before the addition of estrogen (Fig. 1a). Testosterone induced a slight and nonsignificant increase (*p* = 0.15) in colony production. Progesterone also reduced proliferation but not significantly (*P* = 0.74) (Fig. 1a). At the CD235a^+^ stage, estrogen had a marked effect and reduced the proportion of RBCs by at least 6-fold (Fig. 1b). Indeed, we observed a large decrease in the proportion of CD235a^+^ cells (10% vs 56%) (*P* < 0.01) with the addition of estrogen (Fig. 1b). These results suggest that the exaggerated response to hypoxia in CMS cells is governed not only genetically in CMS females but also by the hormonal milieu, as the effect of hypoxia on RBC production is blunted by estrogen. In order to confirm this idea, we first investigated whether male and female cells have similar responses to hypoxia at the cellular level. By analyzing female CMS and non-CMS cells, we made interesting observations. First, postmenopausal CMS and non-CMS cells showed a hypoxic response similar to that of male cells (Fig. 2). In our in vitro model, postmenopausal CMS female cells show an EE phenotype under hypoxia, that is, a phenotypic response similar to that of CMS males. Indeed, CMS cells produced two times the number of BFU-e colonies as non-CMS cells (Fig. 2a). At the CD235a^+^ stage, CMS cells produce about 30-fold higher amount of RBCs as compared to non-CMS cells (Fig. 2b). Second, sex hormones showed a similar response in postmenopausal female cells and in male cells. The addition of estrogen led to a significant reduction in the number of RBCs (Fig. 2c), whereas the addition of testosterone and progesterone did not result in any significant changes.

### The dose and timing of estrogen addition are critical for erythroid cells

**Fig. 3:**
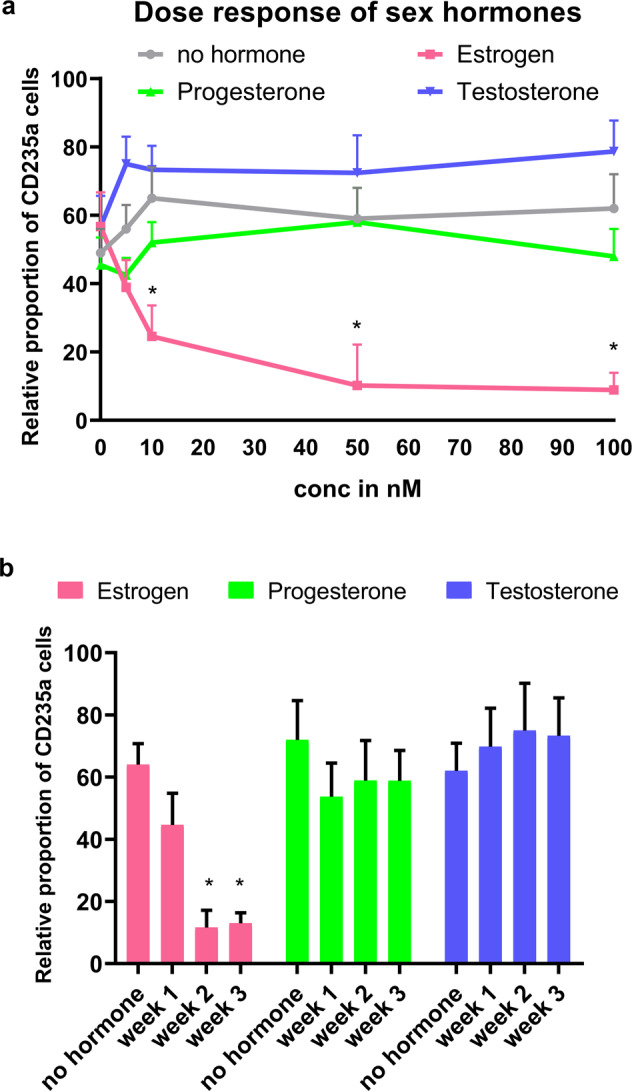
The figure shows the dose response and the stage specificity/effect of the timing of sex hormone addition on male erythroid cells. **a** Different doses (0, 5, 10, 50, and 100 nM) of estrogen, progesterone, and testosterone were used to study the effect of sex hormones on RBC production in CMS subjects. The addition of estrogen above a dosage of 10 nmol resulted in a significant decrease (*P* < 0.01) in RBC production in CMS cells. The line colors depict the following: (i) estrogen (pink), (ii) progesterone (green), (iii) testosterone (blue), (iv) no hormone added (gray). *P < 0.05 (*t* test). **b** Sex hormones were added at various stages during RBC differentiation from iPS cells (at the 1-, 2-, and 3-week EB stages, as shown in the schematic in the “Methods” section) to study the stage specificity of the effect of hormones. Indeed, we observed that estrogen had the strongest impact on RBC reduction when added at the week 2 EB stage. Progesterone or testosterone did not show any specificity or significant effect on the EE phenotype. The bar colors represent the following: (i) estrogen (pink bar), (ii) progesterone (green bar), (iii) testosterone (blue bar), and (iv) no hormone added (first bar in each group). *P < 0.01 (*t* test). The number of subjects in each group is *n* = 3. Data points represent the mean ± SEM for three independent experiments.

It is possible that the dosage or timing of sex hormone addition can change the cell response. To address this, we tested various doses ranging from 5 to 100 nM for estrogen, testosterone, and progesterone (Fig. 3a). Estrogen had a striking negative effect on the erythropoietic response in CMS subjects in a dose-dependent manner (Fig. 3a). At 5–10 nM (physiological estrogen levels: 0.1–10 nM), we observed a significant decrease in RBC production, and the addition of 50–100 nM led to extremely low RBC production. For testosterone, we observed a slight increase, but the changes were not significant (Fig. 3a).

We also tested the effect of hormone addition at different time points during erythroid differentiation on RBC production, as shown in the “Methods” section. iPS cells differentiate into erythroid progenitor cells during each week of cytokine addition until they form mature RBCs, as shown in the schematic in the “Methods” section. For example, at week 1 of the EB stage, the majority of cells are CD45^+^, and at week 2, they are CD71^+^; at week 3, they are CD235a^+^. Therefore, by adding sex hormones at various time points, we investigated the effects of these hormones on specific stages of erythroid precursors. For testosterone and progesterone, we did not observe significant changes (Fig. 3b). However, timing is critical for estrogen. In fact, estrogen showed the strongest RBC reduction in the EB stage at week 2 (Fig. 3b). Since we have shown in our previous study^36^ that there were significant changes in the expression of transcription factors such as GATA1 at week 2 of the EB stage, we hypothesized that estrogen could affect erythropoiesis through specific signaling pathways in CMS and non-CMS cells.

### Estrogen modulates the CMS phenotype via its effects on GATA1 and apoptosis

**Fig. 4:**
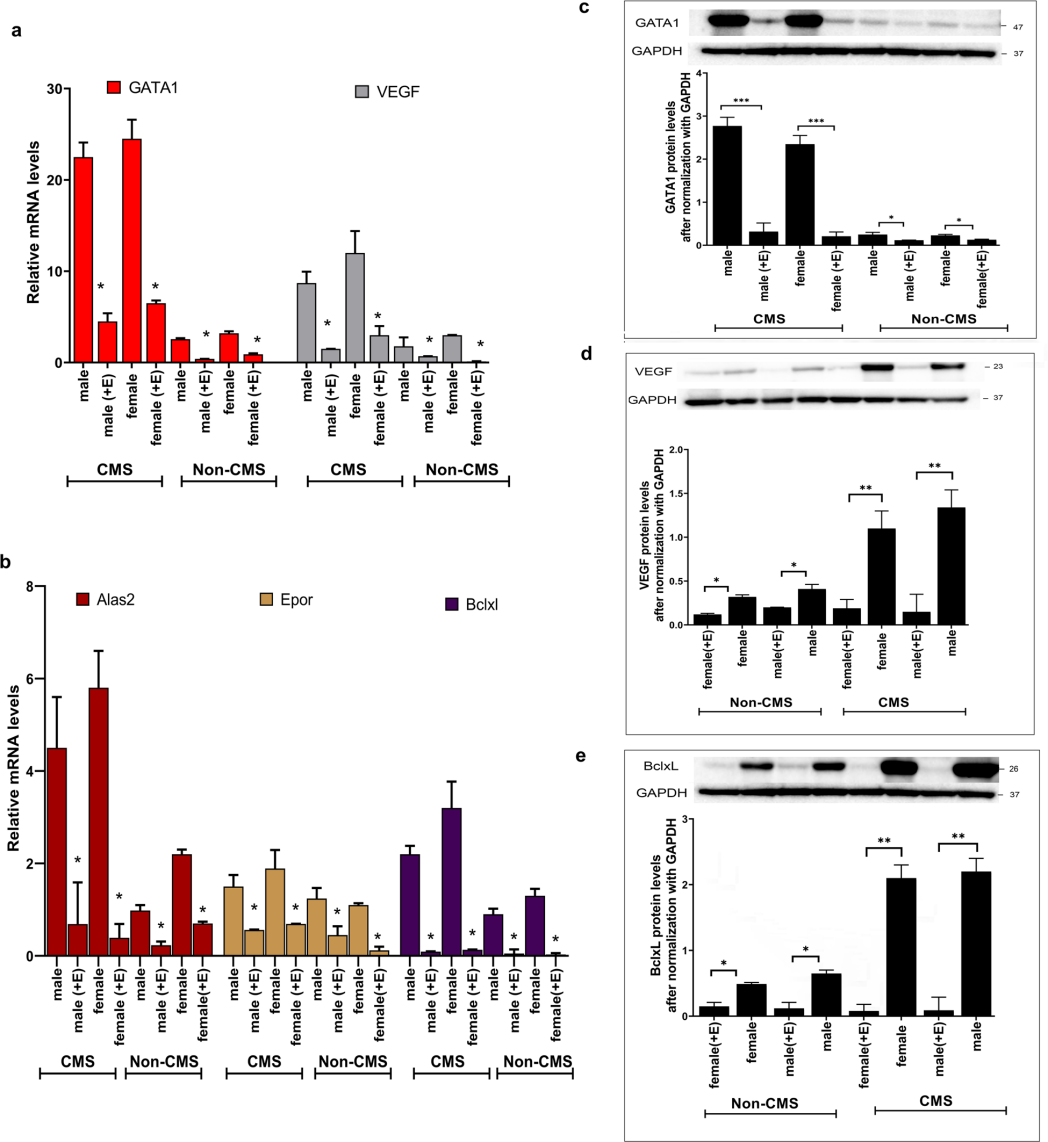
The addition of estrogen significantly reduces the mRNA and protein levels of GATA1, VEGF, and BclxL in CMS (male and female) and non-CMS (male and female) cells. **a** Relative VEGF and GATA1 expression by quantitative PCR (normalized to GAPDH) in cultures grown in a medium under hypoxia. *P < 0.01. **b** Relative GATA1 target gene (*Alas2*, *Epor*, and BclxL) expression by quantitative PCR (normalized to GAPDH) in cultures grown in a medium under hypoxia. *P < 0.001. The number of subjects in each group is *n* = 4. **c** Western blot analysis showing the effect of estrogen on GATA1 protein levels in male and female CMS and non-CMS cells. A representative gel picture is shown on the top, and the summary of the analysis for all the subjects (*n* = 4) is shown below the gel. +E represents the addition of estrogen. CMS and non-CMS samples are marked clearly at the bottom. For each group, male and female samples were tested. The figures show a drastic reduction in the protein levels of GATA1 with the addition of estrogen in both male and female CMS cells. ***P < 0.001. Even for non-CMS cells, the addition of estrogen significantly reduced GATA1 levels in male and female cells (*P < 0.05). **d** Western blot analysis showing the effect of estrogen on VEGF protein levels in male and female CMS and non-CMS cells. A representative gel image is shown on the top, and the summary of the analysis for all the subjects (*n* = 4) is shown below. +E represents the addition of estrogen. CMS and non-CMS samples are marked clearly at the bottom. For each group, male and female samples were tested. The figure demonstrates a reduction in VEGF protein levels with the addition of estrogen in both male and female CMS cells. **P < 0.001. Even for non-CMS cells, the addition of estrogen significantly reduced VEGF levels in male and female cells (*P < 0.05). **e** Western blot analysis showing the effect of estrogen on BclxL protein levels in male and female CMS and non-CMS cells. A representative gel image is shown on the top, and the summary of the analysis for all the subjects (*n* = 4) is shown below. +E represents the addition of estrogen. CMS and non-CMS samples are marked clearly at the bottom. For each group, male and female samples were tested. The figure shows a remarkable reduction in the protein levels of BclxL with the addition of estrogen in both male and female CMS cells. **P < 0.001. Even for non-CMS cells, the addition of estrogen significantly reduced Bclxl levels in male and female cells (*P < 0.05).

**Fig. 5:**
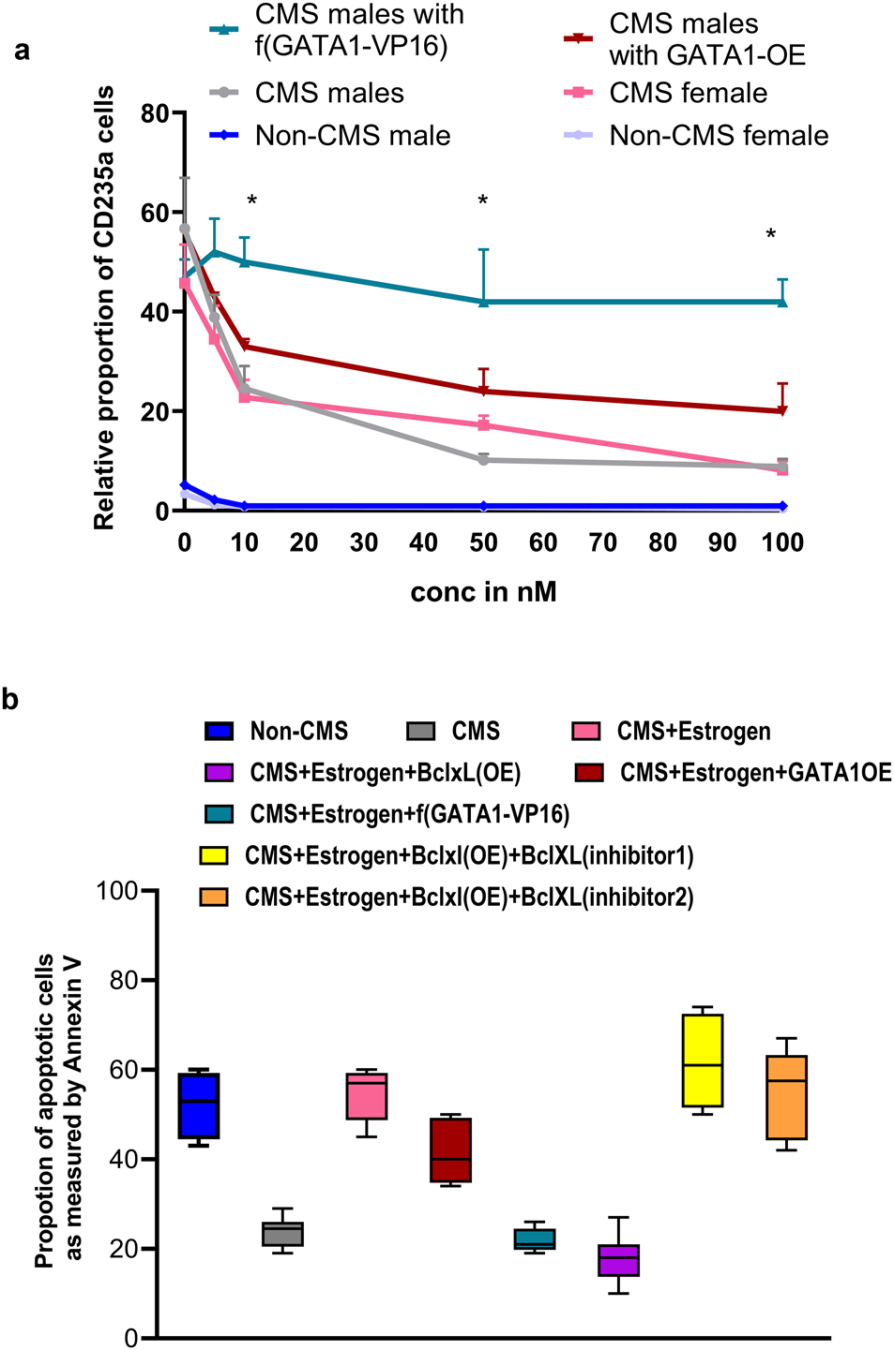
Estrogen-resistant GATA1 OE and BclxL OE are able to reverse the deleterious effect of estrogen on RBC production in CMS subjects. **a** Overexpression of GATA1 (estrogen-resistant f(GATA1-VP-16)) can significantly revert CMS cells to the excessive erythrocytosis CMS phenotype. Non-CMS cells are shown as a control. *P < 0.05 (*t* test). **b** Estrogen increases apoptosis in erythroid cells in CMS cells, which can be prevented by the addition of an estrogen-resistant f(GATA1-VP-16) overexpression construct as well as BclxL OE. Interestingly, the addition of BclxL inhibitors (1 and 2) significantly increased apoptosis. *P* value represents <0.05 (*t* test). The number of subjects in each group is *n* = 3. Data points are the mean ± SEM for three independent experiments.

To identify the signaling pathway by which estrogen regulates erythropoiesis in CMS subjects, we studied the expression of genes that we have shown to be key mediators, such as *SENP1*^36,43^ and *GATA1*^36,43^. Interestingly, we observed that SENP1 levels were similar in CMS males and females before the addition of estrogen, and SENP1 mRNA levels did not change significantly with the addition of estrogen. However, estrogen significantly decreased the mRNA levels of GATA1 and VEGF in cells from CMS males and females (Fig. 4a). VEGF levels decreased approximately 5-fold, and GATA1 levels decreased >10-fold (Fig. 4). Furthermore, the expression of several GATA1 target genes also decreased drastically with estrogen. Figure 4b shows that the expression levels of Alas2, EPOR, and BclxL decreased by ~50%. Furthermore, the addition of estrogen resulted in drastic reductions in GATA1, VEGF, and BclxL levels in both cells from both males and females at the protein level (Fig. 4c–e), reaffirming the critical role of estrogen in regulating them. The effect of estrogen on the expression of GATA1, VEGF, and BclxL was significant even for the non-CMS cells, as shown in Fig. 4. However, the response on the level of RBCs in the CMS cells is remarkable (Figs. 1 and 2), leading us to mainly focus on CMS cells, with non-CMS cells used as controls in each experiment. In order to test the hypothesis that GATA1 is the key downstream target of estrogen, we utilized estrogen-resistant GATA1-[f(GATA-1)-VP16]^45,46^ and studied whether it can eliminate its effect on RBC production. When we overexpressed estrogen-resistant GATA1 and regular GATA1, we found that, unlike wild-type GATA1, estrogen-resistant GATA1 was able to prevent the estrogen-mediated decrease in RBC production in cells from both males and females (Fig. 5a). GATA1 and the GATA1 target gene *BclxL* are critical in regulating apoptosis of erythroid cells. Since estrogen changed the expression of those genes (Fig. 4), we tested the relationship between estrogen and apoptosis in the cells. Indeed, we found that estrogen significantly increased apoptosis in erythroid cells (Fig. 5b). With the addition of estrogen, the apoptosis rate more than doubled (60%) in CMS cells compared to untreated CMS cells (25%). We indeed prevented estrogen-mediated apoptosis by estrogen-resistant GATA1 and BclxL OE (Fig. 5b). We further evaluated the role of BclxL in preventing estrogen-mediated apoptosis by adding specific BclxL inhibitors on the BclxL OE background. Indeed, we observed a drastic increase in estrogen-mediated apoptosis with the addition of inhibitors, suggesting that BclxL plays a critical role in regulating the apoptosis induced by estrogen (Fig. 5b). This strongly suggests that one of the mechanisms by which estrogen modulates erythropoiesis in females is by increasing apoptosis in erythroid cells.

### Estrogen beta signaling is critical in regulating HIF1, VEGF, and GATA1 mRNA levels under hypoxia in CMS individuals

**Fig. 6:**
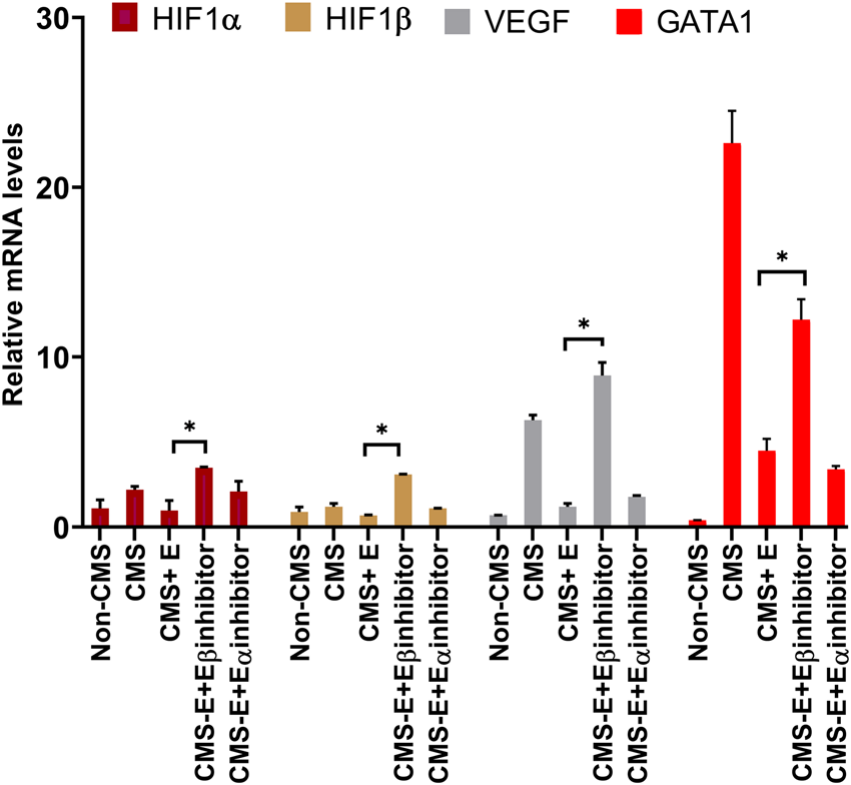
Estrogen beta signaling is critical for the regulation of HIF, VEGF, and GATA1 levels under hypoxia in CMS subjects. The figure shows the expression changes in HIF, VEGF, and GATA1 induced by selectively inhibiting estrogen alpha and beta signaling. *P* value is <0.05 (*t* test). The effects of estrogen on expression are diminished with the addition of an estrogen beta inhibitor, as shown by the increase in the expression of HIF, VEGF, and GATA1. The number of subjects in each group is *n* = 3. Non-CMS cells are shown as a control. Data points are the mean ± SEM for three independent experiments.

In order to further dissect the molecular basis of estrogen effect on the hypoxia-induced EE, we utilized available estrogen alpha- and beta-antagonist and studied their effect on the phenotype. Interestingly, we found that estrogen beta signaling plays a major role in the erythropoietic response of CMS and non-CMS individuals. When we blocked beta signaling, the constraining effect of estrogen on RBC production was abrogated. At the molecular level, we observed that with the addition of the beta-antagonist, the mRNA levels of HIF1 (alpha, beta), VEGF, and GATA1 increased significantly (Fig. 6). With the beta inhibitor, ARNT and HIF mRNA levels were increased by ~2-fold and 1.5-fold, respectively. There was also a major (7-fold) increase in VEGF and an ~2.2-fold increase in GATA1. This strongly suggests that estrogen decreases RBC production through estrogen beta signaling, particularly under hypoxia.

## Discussion

Epidemiological studies have shown sex differences in the prevalence of CMS in the Andean region^7,23^. In addition, the drastic increase in the occurrence of EE in females after menopause reinforces the hypothesis that sex hormones play a role in the etiology of this condition^7,23^. Previous studies have shown a correlation of testosterone and progesterone with hemoglobin levels in CMS patients^24,27–29,47^, but the effect of estrogen on RBC production is still unclear in CMS patients at high altitude. It is possible that all sex hormones have a direct effect on EPO and hepcidin levels and iron metabolism, which directly contribute to the increased hematocrit under hypoxia^48–57^. Physiological factors, such as ventilation, cardiopulmonary responses, and oxygenation, also depend on sex hormones and can also produce differences in hematocrit levels between the two sexes^25,58–65^. Our in vitro model system provides a unique opportunity to study the effect of sex hormones at the cellular level in erythroid cells. In this study, we show a critical role of estrogen in CMS erythroid cells and provide a mechanistic basis for its influence on RBC production.

We have made several important observations in our experiments that show how estrogen regulates hypoxia-induced erythropoiesis at the molecular level. First, estrogen can significantly alter the expression as well as the downstream activity of GATA1 (an essential erythropoietic transcription factor), as reflected by the expression changes of its target genes. In particular, our experiments with estrogen-resistant GATA1 and the rescue of the EE phenotype show a likely direct interaction of GATA1 with estrogen. At high altitude, the effects of estrogen become even more important since our previous study has shown that GATA1 is a central node linked to the pathogenesis of Monge’s disease^36^. It interacts with *HIF1a* and *SENP1*, which are important genes associated with CMS^36,43,66,67^. Therefore, the direct effect of estrogen on GATA1 is an effective mechanism that seems to regulate erythropoiesis in CMS patients. Second, our results show that apoptosis is an important factor in regulating the number of erythroid cells in CMS patients and is directly and causally associated with estrogen. Indeed, the addition of estrogen doubles the apoptotic rates in erythroid cells, and OE of the antiapoptotic gene *BclXL* mitigates its effect on RBCs. Third, the decrease in VEGF expression levels induced by estrogen is also an important finding in this study since VEGF has been previously shown to regulate critical erythropoietic factors such as EPO and GATA1 levels^68–70^. It is possible that VEGF is directly regulated by estrogen or indirectly linked to estrogen through HIF signaling. Fourth, by pharmacological experimentation with specific estrogen receptor inhibitors, we found that estrogen beta signaling is a major pathway by which estrogen regulates RBC production in CMS patients. In fact, inhibiting beta signaling had a direct effect on HIF, VEGF, and GATA1 expression levels. Fifth, the binding of the estrogen receptor is a critical factor in regulating the effect on GATA1. Our rescue experiment in which OE of an ER-resistant GATA-binding protein increased RBC production shows the importance of direct binding. The study by Du et al.^71^ has also showed direct binding of estrogen beta to the GATA1 promoter by chromatin immunoprecipitation and a dual-luciferase assay. In summary, since GATA1 is autoregulated, it seems that the effect of estrogen on GATA1 is the most critical step in estrogen signaling in CMS patients. In our previous study, we showed that SENP1 (a desumoylase) activates GATA1 by removing the SUMO moiety to allow it to enter the nucleus and start its genetic program to produce EE in CMS patients^36^.

Estrogen has been studied in a number of blood diseases at sea level, such as leukemia and anemia^72,73^. Other estrogen-regulated diseases, such as breast cancer, have shown complex regulation and cross-talk between HIF and estrogen signaling^74,75^. In mammals, administration of estrogen leads to severe anemia that has been shown to be caused by apoptosis in erythroid cells^45,46,76^. Mice deficient in ERβ develop chronic myeloproliferative disease during aging, which resembles human chronic myeloid leukemia^77^.

It is interesting to note that maca root, which is a natural herb indigenous to the Peruvian region, is routinely consumed by high-altitude individuals as a potential therapy to lower hematocrit, but the mechanistic basis for its action is unclear^78^. It is possible that maca root interacts with sex hormones, which results in changes in hematocrit. Indeed, a recent study suggested that an increase in estradiol levels occurs after consumption of maca root^79^, but further studies will be required to ascertain its mode of action. From a therapeutic viewpoint, our study is the first step towards understanding the role of estrogen in CMS pathobiology.
